# Data on microsatellite markers in Colletotrichum gloeosporioides s.l., polymorphism levels and diversity range

**DOI:** 10.1016/j.dib.2017.05.012

**Published:** 2017-05-11

**Authors:** Laurent Penet, Sophie Briand, Dalila Petro, François Bussière, Sébastien Guyader

**Affiliations:** INRA, UR1321, ASTRO Agrosystèmes tropicaux, F‐97170, Petit‐Bourg (Guadeloupe) France

**Keywords:** *Colletotrichum gloeosporioides*, Anthracnose disease, Microsatellites, Molecular markers

## Abstract

*Colletotrichum gloeosporioides* is a species complex of fungi belonging to the *Glomerellaceae* family (Ascomycota). It has a global worldwide occurrence and while sometimes described as a plant endophytic commensal, it also often demonstrates pathogenicity on crops and is responsible for anthracnose disease in many cultivated species. Thirty-nine polymorphic microsatellites were isolated and their polymorphism levels were determined in 95 strains from Guadeloupe (Lesser Antilles), mostly isolated from Water Yam (*Dioscorea alata*). The average allele number per polymorphic locus was 12.3 (decreasing to 4.3 at 5% frequency threshold, indicative of dramatic amounts of rare polymorphisms), with a range of 2–29 alleles. The microsatellite markers data will facilitate genetic diversity analyses and population genetics studies for the species complex.

**Specifications Table**

**Table:** 

Subject area	*Biology*
More specific subject area	*Microsatellite markers data (primers and expected diversity levels)*
Type of data	*Table*
How data was acquired	ABI PRISM 3730XL automated sequencer (MACROGEN)
Data format	*Raw (primers information) and partially analyzed (diversity indices)*
Experimental factors	Genomic DNA
Experimental features	*Isolation of microsatellite markers and amplification test*
Data source location	*Guadeloupe 4°44.0694′ N 53°46.881′ W*
Data accessibility	*This manuscript (*Table 1*), primers are also available from probe data bank @ NCBI (*www.ncbi.nlm.nih.gov/probe/*)*

**Value of the data**

• Large set of potentially polymorphic microsatellite markers in *Colletotrichum gloeosporioides.*
• Diversity and genetic structure analyses at both fine and broad geographic scales.
• Pathogenic strains genetic profiling.
• Further *Colletotrichum gloeosporioides* species delineation (complimentary to sequencing data).
• Origin of crop inocula and host origin analyses.

## 1 Data

This dataset is a list of 39 microsatellite markers from the worldwide pathogenic species complex *Colletotrichum gloeosporioides*, including primers and basic information relative to diversity levels expected at each locus. *Colletotrichum* fungi are diversified [1], with species ranging from genuine endophytic commensals to biotrophic parasites or even saprophytic pathogens [2]. Species of this genus are thus often associated with crop diseases, and especially anthracnose in plants [3], [4], [5]. Taxonomic studies are currently investigating sequence based delineation of species (DNA barcoding, e.g. [6], [7], [8]), but reaching consensus is still undergoing [9]. Defining co-dominant and highly polymorphic molecular markers such as microsatellites available for diversity studies and cross geographical or ecological comparisons would be a valuable tool for the study of this species complex and would allow introducing genetic data complementary to the current genomic approaches [9]. Also, these markers might allow differentiating genetic pools that could reflect host adaptation or even possibly identify new species within strain pools (structuration via reduced gene flow, e.g. [6]). We successfully developed 39 microsatellite markers for this wide geographical and ecological range pathogen (Table 1).

**Table 1:** Characteristics for the 39 study microsatellite loci. Probe accession reference can be retrieved at www.ncbi.nlm.nih.gov/probe/. Size range in bp, includes rare alleles of high size. #A is the number of alleles in our 95 strains. F>x% is the number of alleles at a frequency higher than x% in the study sample. Ae is the efficient allele number (1/(1-Nei index)). In bold, 15 loci with amplification levels greater than 80%.

Locus name	Probe accession reference (NCBI)	Repeat motif	Forward Primer 5’ → 3	Reverse Primer 5’ → 3	Amplif. Success	Size range	#A	f>5%	f>1%	Ae	Nei index
Cg12	Pr032825007	tgg	GCAATGGAGCATGCAACTAA	TGGGCTACCTCACATACACG	76%	150–267	10	4	6	4.44	0.77
Cg14	Pr032825015	tcg	TCATTGTCGCCATTTCTACG	GTCTCTGGCGGCTATGTTTC	37%	156–159	2	2	2	1.44	0.31
**Cg16**	**Pr032825021**	**cac**	**ACAAGCAGTTTCTGGCTGCT**	**TGATGATGTCGGTAGGTCCA**	**85%**	**102–276**	**9**	**3**	**7**	**3.41**	**0.71**
Cg19	Pr032825026	gcc	GCGTTGTGAAATTGGACCTC	AGTCTTCAGCGTCAATCCGT	47%	103–224	10	3	6	3.24	0.69
Cg37	Pr032825027	gac	TTGCTGAAGCATACCGTGAG	AAGGTTTGAATTGTGTCGGC	45%	90–103	4	3	3	2.75	0.64
Cg53	Pr032825028	ttg	ACACCAGGAGAAACTCACCG	GGACCAGAACAAGGACCAAA	69%	231–324	14	5	10	7.07	0.86
**Cg57**	**Pr032825029**	**tcg**	**CCGTCTATTGGGGTAGCTGA**	**TGGTCATGGTGCATTTGAAG**	**100%**	**197–239**	**15**	**6**	**12**	**8.20**	**0.88**
**Cg68**	**Pr032825030**	**tcc**	**TGGTCTGCTTCTCGACACTG**	**AGCCAAGAGACCAAGCAAGA**	**87%**	**109–325**	**15**	**5**	**11**	**7.25**	**0.86**
Cg71	Pr032825031	aac	TGATGGTTGTCATGGGATTC	GATCATGTCTCCATCCGCTC	47%	91–250	18	3	9	7.36	0.86
Cg83	Pr032825032	gt	GGATTTGTGCTGTGGGCTAT	GGACAAGAGAATGGAAGGACA	45%	122–218	16	9	16	6.71	0.85
Cg90	Pr032825033	gt	TAGCGTGATCGGAATGCGT	AGTGAATCGAATTGAAGGGC	74%	176–294	12	4	8	5.78	0.83
Cg91	Pr032825034	ga	GGTTGCGACCAATGATCC	GACTCCGGTGAAAATAGCCA	56%	94–136	6	3	6	2.36	0.58
**Cg92**	**Pr032825035**	**tc**	**CATTTTCCACAGCCCACAC**	**GCAGCAGGTGTGAGAAGAGA**	**82%**	**92–250**	**29**	**7**	**13**	**17.3**	**0.94**
Cg93	Pr032825036	tgg	TCTGTGTTGTGATGGTGACG	GCCCGAACCTTCCTCTACTT	45%	86–234	11	6	7	5.45	0.82
**Cg95**	**Pr032825037**	**ca**	**GGAGGTGGTTCGATAGTCGT**	**GGTTCGTTTGACACCACAA**	**80%**	**134–192**	**10**	**4**	**7**	**4.39**	**0.77**
**Cg96**	**Pr032825038**	**ga**	**ACGCGGAGGCATTCAGAG**	**GGAGTCCAATGTTGTGCGTA**	**92%**	**102–258**	**13**	**4**	**7**	**3.29**	**0.70**
Cg97	Pr032825039	at	TTGTTGTGAAAGGAAAGGTTGA	AATCCCACGGGAGAATACAT	41%	112–152	4	2	3	2.11	0.53
Cg98	Pr032825040	tg	CGAGGCAAGCTGTAGCAGTA	TTCGTATTGTCTCCGTTCCC	56%	134–396	19	3	7	9.33	0.89
Cg100	Pr032825002	ag	GATGCATCTCGGGAGACC	CAATTCCCCACGAACATCTC	77%	78–128	9	5	7	4.46	0.78
Cg109	Pr032825003	gt	TCAAAAGACACGACCACGAC	CCATGGATGTGAGCATCATT	74%	130–190	7	3	6	3.94	0.75
**Cg110**	**Pr032825004**	**ac**	**TGATACTGCGATGACAACCG**	**GGCATGGAGAGTCGAACCTA**	**91%**	**94–152**	**8**	**3**	**5**	**3.07**	**0.67**
**Cg115**	**Pr032825005**	**cg**	**CACTGCTAGATGAGGCTTTGA**	**ACAAGTCGAGACGGGAAGAC**	**89%**	**92–182**	**11**	**3**	**5**	**3.06**	**0.67**
Cg116	Pr032825006	ca	CAATCTTATCCCGGCCTTC	GCGGGGTTCAGTCAGAGATA	62%	96–196	15	5	9	8.99	0.89
**Cg120**	**Pr032825008**	**ac**	**AGTTTTCGTCTGAACTGCGG**	**GCGAGCCATAGCCAAAGTAG**	**80%**	**86–176**	**26**	**6**	**16**	**13.2**	**0.92**
Cg122	Pr032825009	ag	CTTTCGGTCAAGGTGTTGTG	CTGGTGCCTCTCAAATCTCC	73%	78–285	17	5	7	7.58	0.87
**Cg127**	**Pr032825010**	**ac**	**GCTTGGTGGTTTAGCCAGTG**	**TTTGCCATATCCATGCTCTG**	**98%**	**208–268**	**10**	**4**	**6**	**3.57**	**0.72**
**Cg129**	**Pr032825011**	**ac**	**GACTCCAGCCACCACAAGAT**	**GATGCCGTTGTACCAGATCC**	**94%**	**72–240**	**18**	**6**	**10**	**5.58**	**0.82**
**Cg131**	**Pr032825012**	**ca**	**GAATGCATTTGGGACGACG**	**AGCCGCTGCAGTTCTTCTAA**	**83%**	**92–160**	**11**	**4**	**5**	**4.02**	**0.75**
Cg136	Pr032825013	gt	AAGTCTCGAGTGGTGAAGGTG	TGCACTGACCTGACTGCTTT	66%	86–194	15	5	9	6.77	0.85
Cg137	Pr032825014	ga	GACGAGGTTGCAAGTCGATA	GGATGGAGGAGTAGAGGCGT	52%	162–262	21	3	9	11.6	0.91
Cg144	Pr032825016	ct	GCCTCCACCATCTATGGACT	GCGACTAGCGTAAGGCAAGA	34%	94–112	8	3	7	5.38	0.81
Cg149	Pr032825017	ga	ACGAGGGAAAATAGGGGATG	CTGCCACTTCGGGTACCTT	78%	86–204	24	5	13	9.38	0.89
Cg150	Pr032825018	gt	TACCAGGGGTGGCAGCTC	GGTCCAGGGACTCAAGCTC	75%	90–232	17	5	11	7.11	0.86
**Cg156**	**Pr032825019**	**gtg**	**ACAGGGACATCCTGTTCAGC**	**CCAGAATCGTCGCTTCAAGT**	**85%**	**87–285**	**15**	**5**	**7**	**4.65**	**0.79**
**Cg159**	**Pr032825020**	**cct**	**GTCCATCTTACCGCGTGTTT**	**CGGTATCAACAACAAACAATCA**	**83%**	**79–91**	**3**	**3**	**3**	**2.21**	**0.55**
**Cg161**	**Pr032825022**	**tacc**	**GGAGAACAGAAAGCGGCG**	**GGATGCGGCTAGTAGGTAAGG**	**88%**	**87–267**	**15**	**4**	**10**	**3.18**	**0.69**
Cg162	Pr032825023	aggt	GCCTTTGTGCTGCATGTAAG	TGCGCAAGATTCACCTACTG	46%	134–150	4	3	4	3.71	0.73
Cg163	Pr032825024	accgc	CACAATACACAATCCACCACG	AGGATGCTATGCAGTCCAGG	62%	142–165	6	5	5	4.35	0.77
Cg164	Pr032825025	ctaca	ACACCGAACAAGCGATCCTA	GACCCTGAAGGCGTGAATAG	49%	283–298	4	3	4	2.91	0.66

## 2 Experimental design, materials and methods

Genomic DNA was extracted from seven strains of *Colletotrichum gloeosporioides*. Six microsatellite-enriched genomic libraries were produced following [10]. DNA was digested with RsaI and fragments of 500 bp were ligated into a pCR 4-TOPO vector. These were then used to transform One Shot TOP10 chemically competent Escherichia coli, producing a total of 1158 positives clones and 128 were sequenced on an ABI PRISM 3730XL automated sequencer, using T3 and T7 primers. Consensus sequences were obtained using ChromasPro 1.34 software [11]. Of these sequences, 21 were of poor quality, 24 did not show microsatellite region, 24 were sister clones, and 59 showed microsatellites (motifs of three repetitions or more). Forty-nine primers pairs were thus designed using Primer-3 [12] and PrimerSelect of DNAStar [13].

The primers were optimized for amplification, testing annealing temperature (44.5–64.2 °C), MgCl2 concentration (1–3.5 mM), and polymerase chain reaction cycles (25–35). PCR conditions consisted of a denaturation stage at 95 °C for 5 min followed by 40 cycles at 95 °C for 30 s, 59 °C for 30 s, 72 °C for 30 s. Thirty-nine loci successfully amplified, all within expected sizes. In a further sample of 95 strains, polymorphism was assessed. High variability in alleles and Nei index were observed (Table 1). We report amplification success in single PCR runs, to help researchers chose loci more specifically. Indeed, *Colletotrichum gloeosporioides* demonstrate high phenotypic plasticity, possibly involving flexible DNA methylation, and amplification might vary depending on methylation state. We thus recommend choosing among these loci with a subsample study first.

In this polymorphism assessment, our strains were sampled from *Dioscorea alata* in Guadeloupe, where anthracnose is the main threat [14] and impacted agro-diversity [15]. Comparisons at wider geographical scales might enlighten important population processes: local dispersal [16], up to migration at greater scales [17], as well as genetic differentiation levels.
